# Oncofertility care for newly diagnosed girls with cancer in a national pediatric oncology setting, the first full year experience from the Princess Máxima Center, the PEARL study

**DOI:** 10.1371/journal.pone.0246344

**Published:** 2021-03-05

**Authors:** M. E. Madeleine van der Perk, Anne-Lotte L. F. van der Kooi, Marianne D. van de Wetering, Irene M. IJgosse, Eline van Dulmen-den Broeder, Simone L. Broer, Aart J. Klijn, A. Birgitta Versluys, Brigitte Arends, Ralph J. A. Oude Ophuis, Hanneke M. van Santen, Alida F. W. van der Steeg, Margreet A. Veening, Marry M. van den Heuvel-Eibrink, Annelies M. E. Bos

**Affiliations:** 1 Princess Máxima Center for Pediatric Oncology, Utrecht, Netherlands; 2 Department of Obstetrics and Gynecology, Erasmus MC–University Medical Center Rotterdam, Rotterdam, Netherlands; 3 Reproductive Medicine and Gynecology, University Medical Center Utrecht, Utrecht, Netherlands; 4 Pediatric Urology, University Medical Center Utrecht—Wilhelmina Children’s Hospital, Utrecht, Netherlands; 5 Pediatric Endocrinology, University Medical Center Utrecht—Wilhelmina Children’s Hospital, Utrecht, Netherlands; Infertility Unit, ASST Lariana, ITALY

## Abstract

**Background:**

Childhood cancer patients often remain uninformed regarding their potential risk of gonadal damage. In our hospital we introduced a five step standard oncofertility care plan for all newly diagnosed female patients aiming to identify, inform and triage 100% of patients and counsel 100% of patients at high risk (HR) of gonadal damage. This observational retrospective study (PEARL study) evaluated the use of this standard oncofertility care plan in the first full year in a national cohort.

**Methods:**

The steps consist of 1)timely (preferably before start of gonadotoxic treatment) identification of all new patients, 2)triage of gonadal damage risk using a standardized gonadal damage risk stratification tool, 3)informing all patients and families, 4)counseling of a selected subset of girls, and 5) fertility preservation including ovarian tissue cryopreservation (OTC) in HR patients using amended Edinburgh criteria. A survey of the medical records of all girls newly diagnosed with cancer the first year (1-1-2019 until 31-12-2019) was conducted.

**Results:**

Of 261 girls, 228 (87.4%) were timely identified and triaged. Triage resulted in 151 (66%) low(LR), 32 (14%) intermediate(IR) and 45 (20%) high risk(HR) patients. Ninety-nine families were documented to be timely informed regarding gonadal damage risk. In total, 35 girls (5 LR, 5 IR, 25 HR) were counseled by an oncofertility expert. 16/25 HR patients underwent fertility preservation (1 ovariopexy + OTC, oocyte cryopreservation (1 with and 1 without OTC) and 13 OTC). Fertility preservation did not lead to complications or delay of cancer treatment in any patient.

**Conclusion:**

We timely identified and triaged most girls (88%) with cancer with a high risk of gonadal damage to be counseled for fertility preservation. We aim to optimize the oncofertility care plan and the standardized gonadal damage risk stratification tool based on this experience and these may be of value to other pediatric oncology centers.

## Introduction

Childhood cancer treatment is accompanied by multiple direct and late toxicities [1]. Preferably, these toxicities are anticipated and ideally prevented already prior to and during cancer treatment. Impaired future fertility is a major concern for childhood cancer patients and their parents. In the past, awareness of infertility as unexpected sequelae was raised only after reaching adulthood [2, 3]. Currently, patients, parents, survivors and healthcare professionals acknowledge the importance of discussing the risk of premature ovarian insufficiency (POI) and consequent infertility due to gonadotoxic cancer treatment [4, 5]. This includes the need for preservation already at an early stage even before the start of cancer therapy [4–8]. It is challenging however, to timely identify patients at risk, to triage and to inform patients of gonadal damage risk on an individual basis before cancer treatment, and to offer the possibility of further counseling for fertility preservation by a fertility expert, without delay of cancer treatment in full cohorts of pediatric patients. The clinical focus upon presentation with new oncologic disease is often on the diagnostic process and swift stratification towards the most effective cancer treatment rather than on preventing potential toxicities. Therefore, our dedicated oncofertility working group created a standardized and easy to apply oncofertility care plan including a gonadal damage risk stratification tool, as this was deemed indispensable to ensure adequate and timely oncofertility care. The aim of the care plan was to identify, inform and triage 100% of all new patients and to timely refer 100% of the girls at high risk (HR) of gonadal damage for expert fertility counseling. This observational retrospective PEARL (PresErving ovARian function through cryopreservation and informing girLs with cancer about infertility due to gonadotoxic treatment) study evaluated the use of this standard oncofertility care plan in the first year 2019 in a full national cohort after centralization of pediatric oncology care.

## Methods

Pediatric oncology care in the Netherlands, previously dispersed in 7 expertise centers, was merged into one national center, the Princess Máxima Center for pediatric oncology in Utrecht in May 2018. This centralization serves the mission to further improve cure rates, while also decreasing early and late toxicity.

### The female oncofertility care plan

**Fig 1:**
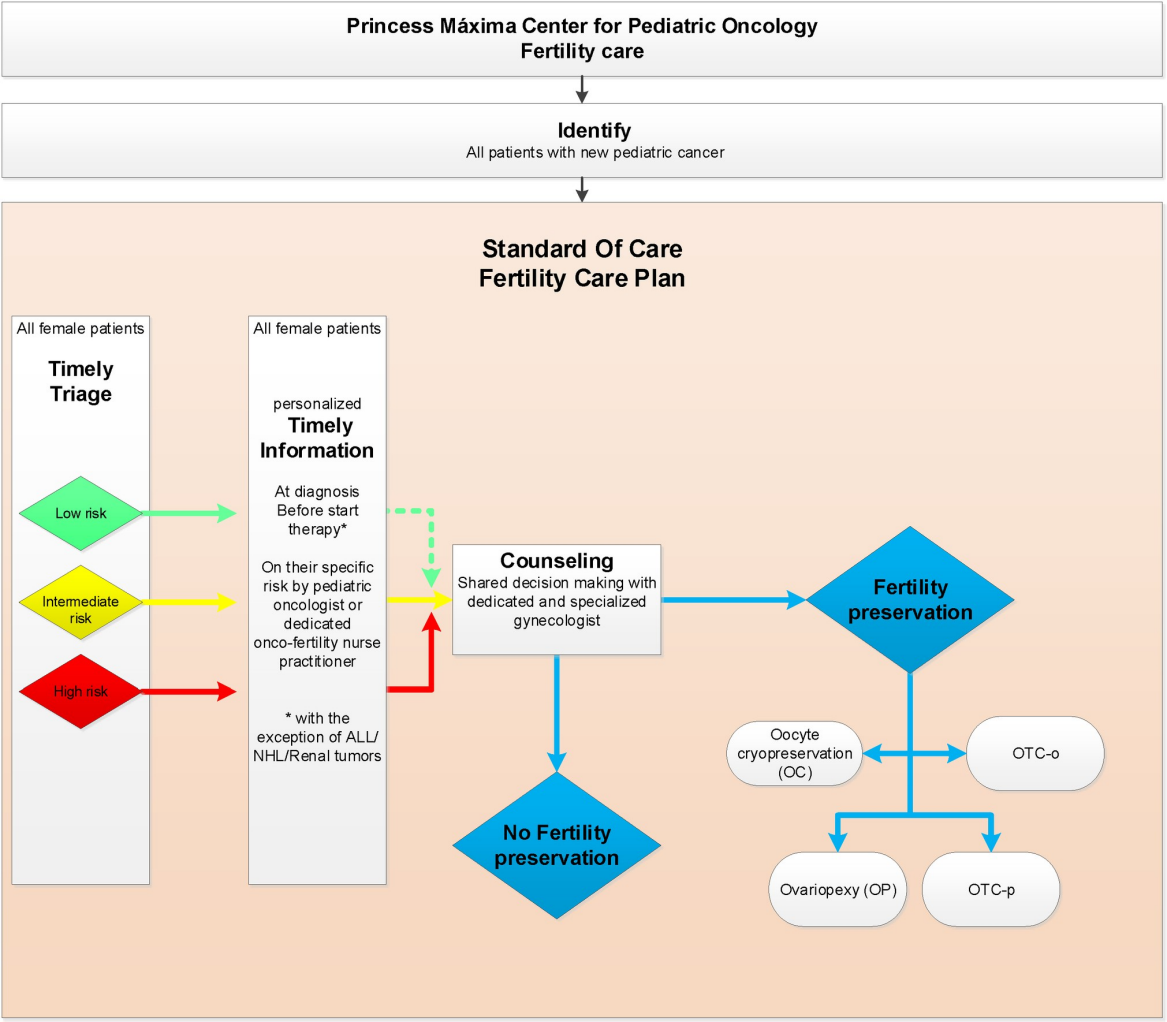
Oncofertility care plan 2019 Princess Máxima Center for newly diagnosed girls with cancer. ALL = Acute Lymphoblastic Leukemia; NHL = Non-Hodgkin lymphoma; OTC-o = ovarian tissue cryopreservation of complete ovary; OTC-p = ovarian tissue cryopreservation of partial ovary.

Since 2015 a multidisciplinary dedicated team with representatives of all departments (S1 Table in S1 Appendix), prepared an oncofertility care plan for newly diagnosed children respecting their right to receive personalized oncofertility care [9]. The oncofertility care plan for newly diagnosed girls (Fig 1) is based on international and national literature and professional experiences [9–12]. The content of our care plan had been intensively discussed with and is approved of by the joined ethical committee of the UMC Utrecht and Princess Máxima Center. This plan includes five steps (S2 Table in S1 Appendix).

#### 1) Identification of all new patients

Identifying newly diagnosed girls with cancer in our center is pursued on a daily basis by a dedicated oncofertility nurse practitioner (coordinator) together with the involved pediatric oncologist. The daily refreshed financial administration, the tumor board lists and clinical ward rounds are used to timely identify new patients.

#### 2) Triage gonadal damage risk

Pursuing standardized triage is done using a gonadal damage risk stratification tool on risk of future gonadal damage. This clinically applicable and easy to use gonadal damage risk stratification tool covers the (most commonly) used European treatment protocols in the Netherlands. As pediatric oncology is a dynamic field, the risk stratification is subject to updates with every new treatment protocol introduced in our hospital and enhancements over time are possible with increasing knowledge. In 2019 the cyclophosphamide equivalent dose (CED) score, expected abdominal radiotherapy (with dose to the ovary), hematopoietic stem cell transplantation (HSCT), and (ovarian) surgery served as the basis premise for this gonadal damage risk stratification tool. In 2019 treatment protocols were classified as LR, IR or HR of gonadal damage with CED scores of 0-4000mg/m2, 4000-8000mg/m2 and more than 8000mg/m2 respectively including radiotherapy and surgery [13–15]. Evidently, other factors such as very young age at diagnosis, prognosis, psychosocial and ethical issues are taken into account upon counseling of patients. The oncofertility plan was mirrored during a working visit to the oncofertility team of the Cincinnati Children’s Hospital, USA, in September 2018 before implementation.

#### 3) Informing patients

Timely (preferably before start of gonadotoxic treatment) all patients are informed on their specific gonadal damage risk by their pediatric oncologist and/or the oncofertility nurse practitioner (coordinator).

#### 4) Counseling a subset of girls by a oncofertility specialist

Timely (before therapy or not leading to delay of therapy) counseling by the oncofertility specialist (gynecologist) is available. In our institute this is available for patients at low risk (LR) and intermediate risk (IR) on request, but actively encouraged in the subset of patients who are at high risk (HR) of gonadal damage.

#### 5) Fertility preservation

Preserving fertility is an option for highly selected, counseled, eligible (HR) patients after shared decision making. Four different methods are available as standard care fertility preservation in our hospital. a) Ovariopexy (OP), which is only useful in girls in whom radiation to the ovary is expected to do significant (and chemotherapy minor or no) damage. b) Oocyte harvest and cryopreservation (OC), after hormonal ovarian stimulation, which is only feasible for oncology patients who are postmenarcheal and when treatment can safely be postponed at least 2 weeks. c) Ovarian tissue cryopreservation (OTC) by unilateral ovariectomy (OTC-o) or d) partial ovariectomy (OTC-p). The care plan offers OTC to all girls newly diagnosed with cancer with high risk of gonadal damage in our center who, in general, are aged 0–18 years at presentation. The DCOG amended-Edinburgh criteria (S3 Table in S1 Appendix) are used to carefully confirm eligibility of girls for OTC in our center [9, 16, 17]. The content of the counseling by the oncofertility gynecologist is described in the S2 Table in S1 Appendix.

### Patients

Only newly diagnosed girls with pediatric cancer between 1 January 2019 and 31 December 2019 were included. The evaluation was conducted in spring 2020.

### Evaluation of the full first year of oncofertility care implementation

Application of the standard oncofertility care plan for newly diagnosed girls and the five step process was evaluated. Baseline data including age at diagnosis, type of malignancy, proposed treatment(-protocol and arm), triage result (gonadal damage risk estimation) and curative or palliative intention of treatment were collected. The date of diagnosis was defined as the date of communicating the cancer diagnosis including the explanation of the intended treatment by the pediatric oncologist with the family as recorded in the patient files. The date of the start of chemotherapy was defined as the starting date of the intended treatment protocol. Date of triage, information provision (pediatric oncologist or oncofertility nurse practitioner (coordinator)) and, if applicable, date and content of the fertility preservation counseling were retrieved from the medical records. Timely was defined as before starting cancer treatment, with the exception of acute lymphoblastic leukemia (ALL) and non-Hodgkin lymphoma (NHL). For these patients triage and informing of the family is generally postponed to the moment of reaching complete remission (CR) or treatment arm allocation. In ALL and NHL patients this moment of complete remission (CR) harbors an added benefit for eventual future use of preserved tissue, because of decreased risk of harboring minimal residual disease or leukemic infiltration. In addition, in renal tumor patients in our center the timely triage and information moment is defined as after surgery when definitive treatment stratification, including optional radiotherapy, is defined.

### Statistical analyses

Descriptive statistics are reported including 95% confidence intervals (CI) for the main findings.

### Ethical approval

Ethical consent was obtained from the METC Utrecht (Medical Ethical Committee Utrecht) and the need for informed consent was waived for this retrospective observational part of the PEARL study (METC research file NL72115.041.19 version 2, METC-protocol number 19/783, Netherlands trial register number NL8192). Information was retrieved from the medical records of all newly diagnosed girls in the Princess Máxima Center in 2019.

## Results

**Fig 2:**
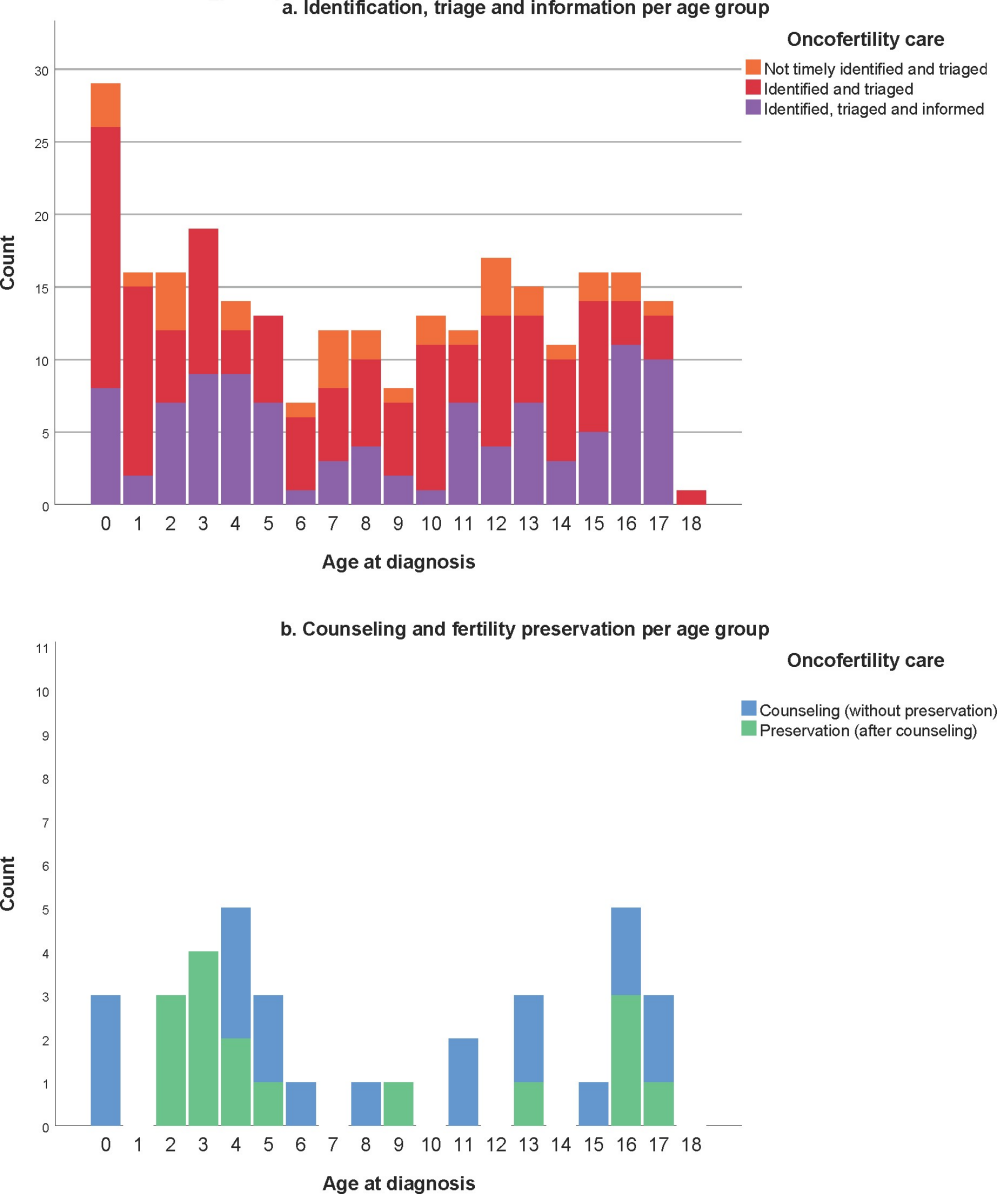
Age at diagnosis and highest received oncofertility care per age group of girls with cancer.

**Fig 3:**
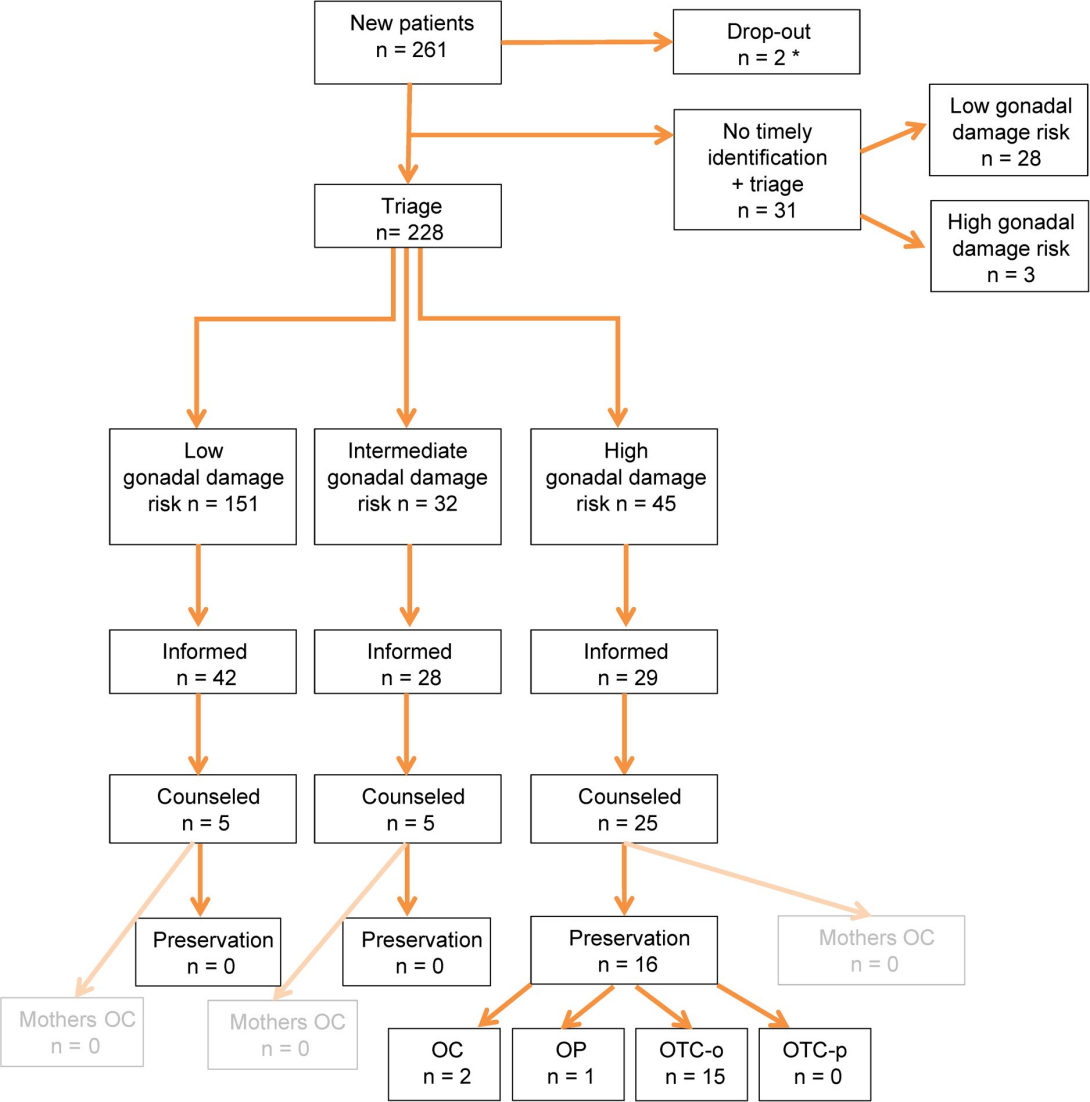
Evaluation of oncofertility care for girls with cancer. OC = oocyte cryopreservation; OP = ovariopexy; OTC-o = ovarian tissue cryopreservation via ovariectomy; OTC-p = ovarian tissue cryopreservation via strips. *2 early deaths occurred in neuro-oncology patients and died prior to triage.

In 2019, 261 girls with a median age of 8.4 years (range: 0.0–18.1) were newly diagnosed with pediatric cancer in the Netherlands (Table 1, Fig 2A and 2B). Two died within days of presentation prior to treatment allocation. Of the remaining 259, 228 (88.0% (95%CI: 0.835–0.914)) patients were timely identified and triaged (Fig 3). Of the 31 patients who had not been timely identified and triaged, 28 were retrospectively classified as LR and 3 as HR. Characteristics of triaged patients seem representative (S4 Table in S1 Appendix). Combining the triaged and the non-triaged patients the 259 patients were classified as LR, IR and HR of gonadal damage in 179 (69.1% (95%CI: 0.632–0.744)), 32 (12.4% (95%CI: 0.089–0.169)) and 48 (18.5% (95%CI: 0.143–0.237)) cases respectively.

**Table 1:** Characteristics of female childhood cancer patients admitted to the Princess Máxima Center in 2019.

	Female childhood cancer patients diagnosed	
	n = 261*	
	n	%
Age at diagnosis (years)** Median (range)	**8.4 (0.0–18.1)**	**-**
Diagnosis		
**Hematologic**	**100**	**38.3**
• Acute Lymphoblastic Leukemia	43	16.5
• Acute Myeloid Leukemia	9	3.4
• Other leukemia’s	3	1.1
• Bone marrow failure/MDS	4	1.5
• Hodgkin lymphoma	23	8.8
• Non-Hodgkin lymphoma	8	3.1
**Solid tumors**	**104**	**39**.**8**
• Neuroblastoma	22	8.4
• Renal tumor	17	6.5
• Carcinoma (hepatic, gynecological)	8	3.1
• Osteosarcoma	6	2.3
• Ewing sarcoma	4	1.5
• Soft tissue sarcoma	11	4.2
• Germ cell tumor	19	7.3
• Skin cancer (incl. melanoma)	3	1.5
• Liver tumors	7	2.7
**Neuro-oncology**	**57**	**21**.**8**
• Brain tumor	57	21.8
**Other** (incl. LCH, Pheochromocytoma etc.)	**17**	**6**.**5**

MDS: myelodysplastic syndrome; LCH: Langerhans Cell Histiocytosis.

*including 2 patients who died premature within days of presentation prior to establishment of treatment

** date at which diagnosis was discussed with patient.

Risk of expected gonadal damage had been communicated timely with parents and child in 99/228 (43.4% (95%CI: 0.372–0.499)) patients out of the aimed 100% of patients to inform. Of these 42/151 (27.8% (95%CI: 0.213–0.354)) were LR, 28/32 (87.5% (95%CI: 0.719–0.950)) IR and 29/45 (64.4% (95% CI: 0.498–0.768)) HR (Fig 3). Reasons for not informing timely triaged patients were available for 125/129 cases, including 14 HR patients. Poor prognosis (n = 13), low risk of gonadal damage (ALL n = 19, renal tumors n = 11, resection only n = 28, other n = 20), psychosocial issues (n = 9), wait-and-scan regimens: no therapy (n = 14) and palliative setting at diagnosis (n = 3) were recurrent reasons for not informing these timely triaged patients (Table 2). Two patients in the HR group were initially classified as LR with resection only. However, after treatment intensification their risk shifted to HR and subsequently these patients had unfortunately not been informed again. Of 42 LR timely informed patients, 5 families requested and received further counseling by a fertility expert. Fertility preservation was not advised nor pursued in these 5 patients.

**Table 2:** Reasons patients were *not* informed after triage.

	Low gonadal damage risk	Intermediate gonadal damage risk	High gonadal damage risk	Total
	n = 109	n = 4	n = 16	n = 129
Poor prognosis	10	0	3	13
Low risk	20	0	0	20
Perspective of the oncologist: too burdensome	1	0	2	3
Family and psychosocial issues*	3	1	5	9
Palliative treatment	3	0	0	3
ALL LR	19	0	0	19
Renal tumor LR	11	0	0	11
Resection only LR**	28	1	2	31
Young age***	0	0	1	1
Long PICU admission in diagnostic phase	0	0	1	1
Wait and scan policy: no treatment	14	0	0	14
Unknown ****	0	2	2	4

ALL LR: Acute Lymphoblastic Leukemia at low risk of gonadal damage; LR: low risk; PICU: Pediatric Intensive Care Unit.

* In some situations the pediatric oncologist sensed that extra information other than the necessary treatment information would be too burdensome for parents. Some patients had very complex family situations.

** Two patients in the high risk group were initially classified as low risk with resection only, but after a change in treatment this risk shifted to high risk and patients were not subsequently informed.

*** This patient was 6 months old at diagnosis.

****Unknown consist of one patient with a medulloblastoma and high risk of gonadal damage receiving ACNS0332. The oncologist of one patient with a neuroblastoma stage 4 decided that fertility information at that time was not applicable and later when the treatment protocol changed to a high risk of gonadal damage no fertility information was provided.

Of 45/228 (20%) triaged HR patients, 29 families had received personalized information. The reasons why 16 HR patients did not receive information after triage are depicted in Table 2. In all 29 informed HR cases counseling by a dedicated oncofertility gynecologist was strongly advised and 25 (86.2%) (of the aimed 100% counseled HR patients) appreciated such counseling. The 4/45 HR families that did not wish referral for counseling felt that they had been sufficiently informed (three were eligible for preservation, but did not wish preservation or counseling and one girl was very young (<6 months)).

In 16 out of the 25 HR girls counseling led to an attempt to preserve fertility (Table 3). One patient underwent the combination of OC+OTC-o (n = 1). This patient initially had time to delay treatment for oocyte harvest. However, due to the limited number of harvested oocytes and insufficient time to perform another cycle, additionally OTC-o was performed. In five patients fertility preservation was performed prior to the start of cytotoxic treatment, eleven preserved gonadal tissue during treatment (S1 Fig in S1 Appendix). No intra-operative or post-operative complications, such as wound infections, bleeding requiring transfusion, reoperation, ICU admittance or mortality were reported. Reasons for not preserving ovarian tissue in HR patients mainly included uncertainty about the success of future auto-transplantation or use of the tissue (Table 4). The option of OC in young mothers (<40 years) of pediatric cancer patients for future oocyte donation was mentioned during counseling (S2 Table in S1 Appendix). In 2019 none of the mothers pursued this option.

**Table 3:** Girls in which active fertility preservation intervention was pursued.

	Fertility preservation in HR group
	n = 16
**Age at preservation (years)** Median (range)	4.6 (2.5–17.5)
**Malignancy type**	
Hematological *	4
Solid **	12
Neuro-oncology	0
**Type of preservation**	
OTC-o (complete ovariectomy)	13
OTC-p (partial ovariectomy)	0
Oocyte cryopreservation (OC)	1
OC + OTC (complete ovariectomy) ***	1
Ovariopexy (OP) + OTC (complete ovariectomy)	1

HR: high risk; OTC = Ovarian Tissue Cryopreservation.

* The hematologic malignancies included 1 patient with Acute Lymphoblastic Leukemia–HR protocol, 1 Acute Lymphoblastic Leukemia stem cell transplantation (SCT), 1 Acute Myeloid Leukemia SCT and 1 Myelodysplastic syndrome SCT.

** The solid tumors included 5 patients with neuroblastoma, 1 ovarian carcinoma, 3 Ewing sarcomas, 2 soft tissue sarcoma and 1 bilateral metastasized Wilms tumor receiving stem cell transplantation.

*** This high risk patient initially had the time to delay treatment for oocyte harvest. However due to the limited number of harvested oocytes and no time to perform another cycle, it was decided to additionally perform OTC.

**Table 4:** Reasons why patients did *not* desire to preserve fertility after counseling.

	Frequency	%
Low or intermediate gonadal damage risk (LR, IR)	10	3.9
No guarantee of success auto-transplantation (HR)	4	1.5
Too burdensome on the child, preference for oocyte cryopreservation after age 16 (HR)	2	0.8
The risks do not outweigh the benefits and genetic parenthood is not the most important (HR)	1	0.4
Fertility is no issue due to genetic mutation (HR)	1	0.4
Preference for oocyte cryopreservation at 16 years (HR)	1	0.4
Total	19	7.3

## Discussion

This retrospective study in a national cohort evaluated our first year of centralized oncofertility care in girls with cancer. It aims to search further improvement of quality of care. In the first full year this risk stratification seems to have led to timely identification and triage of 87.4% of all newly diagnosed girls with cancer using a protocol(-arm) based oncofertility gonadal risk stratification tool (Table 5). Fertility preservation in 16 patients did not cause delay in the onset of oncological treatment. Of the 31 not timely identified and triaged patients, 28 were LR and three were HR patients. Hence, although improvement is necessary, identification of most HR patients seems feasible.

**Table 5:** Gonadal damage risk stratification tool for European treatment protocols used for oncofertility care for girls in the Princess Máxima Center.

Tumor	Protocol	Treatment arm	CED mg/m2	Female gonadal damage risk
Hematologic malignancies				
Acute Lymphoblastic Leukemia*	ALL-11	SR, MR	2000	Low
		HR 1–3 +SCT	5600 + SCT	High
		HR 1–6 + II	9300	High
	Interfant06	Germline LR/rearranged MR HR—SCT	3000	Low
		rearranged MR HR + SCT	3000+ SCT	High
	EsPhALL	Arm A	9000	High
		Arm B	3000	Low
		High risk arm	3976 + SCT	High
		High risk arm	5976 + SCT	High
	IntReALL	SR treatment arm A	1976	Low
		SR treatment arm A with SCT	1976 + SCT	High
		SR treatment arm B	3400	Low
		SR treatment arm B with SCT	3400 + SCT	High
		HR	1976 + SCT	High
	ALLTogether**	R1 standard, experimental	0	Low
		R2 standard, Exp arm A, Exp arm B	3000	Low
		R3 standard, Exp InO: IR-high risk	2000	Low
		ABL HR allo-SCT (≥ 1–3 NOPHO blocks)	2000 +SCT	High
		ABL IR-high	2000	Low
		HR BCP SCT 3 blocks	4200 +SCT	High
		HR BCP chemo 6 blocks	7400	Intermediate
		HR T-cell without Nelarabine + HR blocks	4200	Intermediate
		HR T-cell with Nelarabine single	1000	Low
		HR T-cell with Nelarabine single + HR blocks	3200	Low
		HR T-cell with Nelarabine addition	2000	Low
		HR T-cell with Nelarabine addition + HR blocks	4200	Intermediate
		DS-SR	1000	Low
		DS-IR, DS-HR	3000	Low
LCH	LCH IV	stratum1 group 1 (MS-LCH) arm A / B / C / D	0	Low
(Langerhans Cell Histiocytosis)		stratum 1 group 2 (SS-LCH)	0	Low
		stratum 2	0	Low
		stratum V without clinical neurodegeneration	0	Low
		stratum V with clinical neurodegeneration	0	Low
Hodgkin lymphoma *	EuroNet-PHL-C2	TL1	1000	Low
		TL2	2000	Low
		TL2 intensified	2500	Low
		TL3	4000	Intermediate
		TL3 intensified	5000	Intermediate
Non-B NHL (Non-Hodgkin	Euro LB-02	T-Cell LL stage I-II	2000	Low
Lymphoma)		T-Cell LL stage III-IV	3000	Low
		non-T-Cell LL stage I-II	2000	Low
		non-T-Cell LL stage III-IV	3000	Low
B-NHL/B-ALL *	SKION B-NHL/B-ALL	Group A	3000	Low
(B-cell Non-Hodgkin Lymphoma/	2008	Group B	3300	Low
acute lymphoblastic leukemia)		Group C1	6800	Intermediate
		Group C2	6800	Intermediate
	Inter-B-NHL ritux	Group B HR	3300	Low
		Group C1	5800	Intermediate
		Group C3	5800	Intermediate
		PMLBL	4500	Intermediate
Anaplastic Large Cell Lymphoma	ALCL	LR	3352	Low
		SR arm 1 SR arm 3, HR arm 1, HR arm 2, HR arm 3, HR arm 4	6328	Intermediate
Acute Myeloid Leukemia *	Nopho DBH AML		without SCT	Low
	2012		with SCT	High
Acute Promyelocytic	ICC APL 01	SR MRD- / SR MRD+ / HR	0	Low
Leukemia	ICC APL 02	SR, HR	0	Low
**Solid tumors**				
Neuroblastoma *	DCOG NBL 2009	OG without N4	0	Low
		OG with 1x N4	2100	Low
		OG with 2x N4	4200	Intermediate
		OG with 3x N4	6300	Intermediate
		OG with 4x N4	8400	High
		MR without N4	10290	High
		MR with N4	18690	High
		HR without N4	12690	High
		HR with N4	21090	High
	DCOG NBL 2009 <1yr	OG with 1x N4 <1yr	/kg	Low
		OG with 2x N4 <1yr, 3x N4 <1yr, 4x N4 <1yr	/kg	Intermediate
		MR without N4 <1yr, with N4 <1yr	/kg	High
		HR without N4 <1yr, with N4 <1yr	/kg	High
Ewing*	Ewing 2008	R1 female	25176	High
		R3	25176	High
		R3 + TreoMel	30776	High
Osteosarcoma*	EURAMOS 1	MAP	0	Low
		MAPIE	14640	High
Renal tumors*	UMBRELLA 2016/SIOP 2001***	AV + AVD, AV + AV1, AV + AV2	0	Low
		AV + HR	8100	High
Rhabdoid tumors *	EpSSG NRSTS 2005	Cyclophosphamide	17000	High
of the kidney	EURHAB <18 mo	3x DOX, 3x ICE, 3xVCA	8892	High
(RTK) or of soft	EURHAB <18 mo HD	2x DOX, 2x ICE, 2x VCA + CARBO Thiotepa	50928	High
tissue (MRT)	EURHAB >18 mo	3x DOX, 3x ICE, 3xVCA + RT	8892	High
	EURHAB >18 mo HD	2x DOX, 2x ICE, 2x VCA + CARBO Thiotepa + RT	50928	High
NRSTS*	EpSSG NRSTS 2005	3x ifosfamide	6588	Intermediate
(Non-Rhabdomyosarcoma Soft		4x ifosfamide	8784	High
Tissue Sarcoma)		5x ifosfamide	10980	High
		6x ifosfamide	13176	High
Soft tissue sarcomas*	EpSSG	LR subgroup A	0	Low
	RMS2005	SR subgroup B	5800	Intermediate
		SR subgroup C (9x Ifosfamide)	13176	High
		SR subgroup C (5xIfosfamide)	7320	Intermediate
		SR subgroup D (9x Ifosfamide)	13176	High
		HR and group A + group C	13176	High
		HR and group A + group D	17376	High
		HR and group B + group C	13176	High
		HR and group B + group D, VHR	17376	High
Germ cell tumor	SIOP CNS GCT II	NGGCT	7320	Intermediate
Liver tumors	PHITT	group A1 very low risk HB	0	Low
Hepatocellular		group A2 very low risk HB	0 (cisplatin)	Low
carcinoma		group B1 Low risk HB / B2	0 (cisplatin)	Low
		group C intermediate risk SIOPEL3HR / C5VD/ CDDP-M	0 (cisplatin)	Low
		group D1 high risk HB SIOPEL4, D2 high risk HB CDCE, CDVI	0 (cis/carboplatin)	Low
		group E1 resected HCC	0	Low
		group E2 resected HCC PLADO	0 (cisplatin)	Low
		group F unresected/metastatic PLADO sorafenib, GEMOX	0 (cisplatin)	Low
**Brain tumors**				
Opticus glioma	SIOP LGG 2004	Vincristine, carboplatin, etoposide. (In case of allergy: cyclo)	0	Low
Intradural-extramedulary tumor	HIT-MED + SCT		49500	High
Medulla blastoma	SR ACNS0331	Cyclophosphamide, lomustine	13200	High
	HR ACNS0332	cyclophosphamide	12000	High
AT/RT (Atypical	EURHAB <18 mo	3x DOX, 3x ICE, 3xVCA	8892	High
teratoid/rhabdoid	EURHAB <18 mo HD	2x DOX, 2x ICE, 2x VCA + CARBO Thiotepa	50928	High
tumors)	EURHAB >18 mo	3x DOX, 3x ICE, 3xVCA + RT	8892	High
	EURHAB >18 mo HD	2x DOX, 2x ICE, 2x VCA + CARBO Thiotepa + RT	50928	High
Dysgerminoma	WHO IV SIOP CNS GCT II HR-non-germinoma	PEI	8540	High
High grade glioma, Pons glioma	ACNS0126	Temozolamide	0	Unknown
Medulloblastoma	PNET 5	MB-SR / MB-WNT-HR(>16years)	17600	High
		MB-WNT-HR (<16years)	13200	High
		MB-SHH-TP53: No alkylating agents	0	Low
HGG (High grade glioma)	Infant HGG 2013/HIT SKK	Elements IIs IIIs/1 IIIs/2 IVs	7200	Intermediate

*Total Body irradiation, full abdominal/pelvic radiation upgrades towards high risk. Expected unilateral removal of an ovary as part of the oncologic treatment in combination with gonadotoxic chemotherapy is also classified as high risk and OTC must be discussed.

** The ALLTogether protocol was not used in 2019. But in anticipation of the starting study, this was already included in this overview.

***The UMBRELLA protocol was initiated in 2019 an prior to that the SIOP-2001 protocol was used, so both protocols were included.

To ensure timely provision of information on potential gonadal damage to all patients, we experienced the importance of daily and central coordination of identification and triage. For that purpose we built a logistic administrative system. We learned that appointing a dedicated oncofertility nurse practitioner who coordinates the navigation of all newly identified patients in the hospital is of utmost importance. Subsequently, patients can be navigated through the oncofertility triage system in due time, mostly before starting cancer treatment and in close communication with the multidisciplinary team (S2 Table in S1 Appendix). Triage can be a complicated effort, which lies just outside the main priorities and expertise of pediatric oncologists. Therefore, we developed a standardized gonadal damage risk stratification tool, based on international protocol(-arms), used at that time in our country. This was deemed instrumental (Table 5). This tool improved over time and the most recent, currently used gonadal damage risk stratification tool is available in the S5 Table in S1 Appendix.

We identified and triaged 82.2% of all girls aged 13 years and older and timely informed 49.3% of them (Fig 2a). Unfortunately, our retrospective study shows that 16 HR patients had not been informed after triage. Several reasons were reported such as very poor expected outcome, serious (co-)morbidity, psychosocial challenges and young age (Table 2). The three clinical practice guidelines of the American Society of Clinical Oncology [10–12] contain evidence-based recommendations for fertility preservation for patients with childhood cancer. A study of compliance with these guidelines reported that none of the 136 patients above the age of 13 had been counselled for fertility preservation [18, 19]. This illustrates how difficult the oncofertility care logistics process can be in real life clinical practice. Evidently, the IR and HR groups are the most relevant group to inform timely on their gonadal damage risk, as these patients might be eligible for fertility preservation. Nevertheless, providing information also to LR patients has been shown to be of value for survivors and family [20]. Previous surveys have shown that many patients and families worry about future infertility at diagnosis already [20–23]. Compared with available literature our percentage of informed patients is reasonably high, even though, we did not reach our aimed 100% informed patients. More effort is needed in the coming years to ensure that all patients with an acceptable cure rate are at least informed about their fertility. Preferably, HR patients will also be referred for counseling to a fertility expert (gynecologist) to explore the opportunities of fertility preservation.

We learned that as treatment is sometimes intensified (n = 2), gonadal damage risk may increase and patients may need to be re-triaged, re-informed and that counseling may need to be reconsidered. For that purpose, presence of an oncofertility nurse practitioner (coordinator) at the multidisciplinary tumor board can enhance awareness in the oncology team. Why a large proportion (mainly LR) was not timely informed was not always documented. Hence, we learned it is important to facilitate a standard documentation process in the summary part of medical records stating whether oncofertility information is provided, and if not the reason why. This is now standard in our current practice. This is consistent with the recently published consensus of the international guideline harmonization group (IGHG), which stated that all childhood cancer patients and their families have the right to be informed regarding their gonadal damage risk [24–26].

As indicated, adjustments have already been made in our current standard care oncofertility plan. We integrated recent recommendations from the IGHG guideline that classifies a CED score of 6000mg/m2 as high risk instead of the 8000mg/m2 which we used in 2019 in our gonadal damage risk stratification tool (S5 Table in S1 Appendix) [24–26]. Additionally, low risk does not mean no risk, as patients with a very low risk may experience infertility after cancer treatment [27]. We should take into account that individual susceptibility and genetic variation may also influence individual risk of gonadal damage [27–29]. The optimal moment to provide information regarding gonadal damage risk has been discussed in our oncofertility working group. Previous studies showed that for both patients and parents the preferred moment is at diagnosis, before cytotoxic medication is applied [2, 20–22]. This corresponds to our aim to inform all girls at diagnosis as intervention is still feasible then. However, we discovered that for female ALL and NHL patients the moment of triage and information provision can be postponed to the moment of CR or treatment arm allocation. We amended this early in the implementation phase of the oncofertility care plan. In addition, over time, for children with renal tumors (with the exception of full blown ruptured patients [30]), we postponed the information process until after surgery (4–6 weeks), as the final gonadotoxic treatment stratification takes place based on histological stage and subtype. More recently, we learned that choosing the moment of ovarian preservation in patients with large abdominal tumors, such as neuroblastoma, 3–6 weeks into treatment may be beneficial for surgical and safety reasons, despite the adverse gonadotoxic influence of 1 or 2 courses of chemotherapy.

Even though all patients may request additional counseling by experts, we learned this opportunity is not always utilized. Of 45 HR patients only 25 were counseled and of these 25 patients, only 16 chose to preserve gonadal tissue. OTC-o was the most common procedure. Oocyte cryopreservation before cancer treatment was no option for most girls in our cohort due to young age and/or lack of opportunity to delay oncologic treatment (Table 4 and S1 Fig in S1 Appendix). As no adverse events occurred, we consider OTC a safe procedure although our numbers are obviously limited. From previous reports, only limited information is available on the safety of OTC [31]. In 2019, we chose not to not perform OTC-p to avoid previously reported bleeding risks and as the majority of our population was very young with small ovaries [31]. Although age is no absolute contra-indication for OTC, we are hesitant to perform OTC in children under the age of 1 year in our center based on the published suggested potential higher anesthesia risk in infants [32]. So far, we did not perform ovarian tissue cryopreservation in infants under the age of 1 year. Nevertheless, evidence for this anesthesia risk in laparoscopic procedures is not strong [32–35]. The risk of gonadal damage will therefore always need to be weighed against the risk of direct toxicity for individual patients. Thus to infants who will, with no doubt, receive high dose HSCT (e.g. Juvenile myelomonocytic leukemia (JMML)) or high dose total-abdominal radiotherapy (e.g. after extensive rupture at presentation in renal tumor or neuroblastoma patients), counseling will be offered and OTC seriously considered.

Decisions to perform OTC were always based on shared decision making. One of the main reasons for deciding against the OTC option was the communicated uncertainty of success of future auto-transplantation (Table 3). Although studies in adults have shown promising results, ex-vivo maturation of ovarian material harvested during childhood is still not pursued. Future auto-transplantation of ovarian tissue from children is still considered experimental, in contrast to OTC, which is now considered standard care [14, 36]. This is explicitly explained to patient and parents during fertility counseling in our hospital (S2 Table in S1 Appendix). Future research on auto-transplantation of ovarian tissue harvested in prepubertal girls will shed more light on the effectiveness of, and may lead to more patients opting for, OTC in the future [17]. Alternatively, OC after finalizing cytotoxic treatment is a feasible option for patients older than 16 years. This can be done starting 1 year after the end of treatment and with a sufficient ovarian reserve. However, there is substantial evidence that patients with excessive doses of alkylating agents, local irradiation, or following HSCT already have diminished ovarian reserve. It is conceivable that they may not benefit from such procedures [37–40]. Thus, also for these patients preventive strategies at diagnosis, as used in our oncofertility plan, have a higher chance of creating fertility options in the future.

Even though most young mothers are informed about the option of OC, none pursued this option in 2019. This may be influenced by the fact that the costs are not covered by insurance companies, as they qualify as “social freezing”. However, we did not investigate the reasons in this retrospective study. When this option is pursued the oocytes are stored under the mother’s name to prevent children to feel obliged to use oocytes of the mother. When age permits, mothers are advised to freeze their oocytes after the end of cancer treatment of their daughter and thus at a less stressful time.

To improve our oncofertility care, the prospective part of the PEARL study currently evaluates the oncofertility care from a patient and parent point of view. It will explore in depth the reasons to preserve or not and the effect of ovarian tissue cryopreservation on the ovarian reserve. Patients’ and parents’ recall of fertility information in cancer survivors is known to be limited [41]. We also aim to evaluate whether information provided by the pediatric oncologist or the dedicated nurse practitioner (coordinator) is deemed sufficient by LR and selected IR patients. The prospective study will further analyze whether the provided information is consumed, comprehended, and still remembered at the moment of discontinuation of therapy. Furthermore, we will analyze whether an information moment at the end of treatment would be a welcome addition to the quality of care of individual patients. In the prospective study the effectiveness of a protocol(-arm) based oncofertility gonadal risk stratification tool will also be evaluated.

## Conclusion

Our study suggests that it may be valuable and clinically feasible to timely identify and triage 87% of all newly diagnosed girls using a protocol(-arm) based oncofertility gonadal risk stratification tool. OTC seems a safe procedure and shared decision making led to a highly selected subgroup of patients for OTC. However, the safety of OTC needs to be confirmed in large prospective studies. In our center implementing oncofertility care did not cause delay in the onset of cancer treatment. We will continue to use the adjusted oncofertility care plan and evaluate this in the prospective PEARL study which started in 2020. We hope that our oncofertility care plan and risk stratification tool may be of use to other pediatric oncology institutes.

## Supporting information

S1 Appendix (DOCX)
