# Supplementary material for: Oncofertility care for newly diagnosed girls with cancer in a national pediatric oncology setting, the first full year experience from the Princess Máxima Center, the PEARL study
Source: PLoS One. 2021 Mar 5;16(3):e0246344. doi: 10.1371/journal.pone.0246344 (PMC7935241; doi:10.1371/journal.pone.0246344)
Supplement: S1 Appendix — (DOCX) [file pone.0246344.s001.docx]

**S1 Appendix.**

Oncofertility care for newly diagnosed girls with cancer in a national pediatric oncology setting, the first full year experience from the Princess Máxima Center, the PEARL study.

**Contents**

S1 Table. Oncofertility taskforce ……………………………………………………………………………………2

S2 Table. How to set up a fertility counseling service (including counseling content) ..………………………….3

S3 Table. DCOG amended version of the Edinburgh selection criteria for OTC ...……………...……………….…..5

S4 Table. Characteristics of timely triaged girls organized per risk group ………………………………...…..6

S5 Table. Updated Gonadal damage risk stratification tool for European treatment protocols used for oncofertility care for girls in the Princess Máxima Center ..…………………………………………………………….…7

S1 Figure. Days between start of chemotherapy and fertility preservation in patients who underwent fertility preservation …………………………………………………………………………………………………..…10

### **S1 Table. Oncofertility taskforce**

| **Specialty** | **Number of representatives** |
| --- | --- |
| Dedicated pediatric oncologist Hemato-oncology/stem cell transplantation | 2 |
| Dedicated pediatric oncologist Solid tumors | 1 |
| Dedicated pediatric oncologist Neuro-oncology | 1 |
| Dedicated oncofertility nurse practitioner | 1 |
| Oncofertility gynecologists | 2 |
| Pediatric urologist | 1 |
| Pediatric endocrinologist | 1 |
| Pediatric oncology surgeon | 1 |
| Clinical embryologist | 1 |
| Child-development specialists | 2 |
| Pediatric psychologist | 1 |
| Social worker | 1 |
| Adult late effects specialist | 1 |

**S2 Table. How to set up a fertility counseling service**

| **Oncofertility care plan step:** | **Relevant medical staff** | **Tasks** |
| --- | --- | --- |
| 1: Identification | - Oncofertility coordinator^ together with responsible pediatric oncologist | - Identification of new cancer patients |
| 2. Triage# | - Oncofertility coordinator^ together with the dedicated pediatric oncologists per department (S1 Table) (in connection with the tumor board) and fertility expert | - Triage of gonadal damage risk  - Arrange logistics of informing families |
| 3. Provision of information## to families | - Pediatric oncologist and/or oncofertility coordinator^  - Psychosocial support | - Timing and provision of gonadal damage risk information.  - Documenting of conversation in medical records  - Supporting the patient and family during the process.  - When applicable: arranging logistics of expert counseling (HR: advised, LR and IR: allowed) |
| 4. Counseling*# | - Fertility expert (gynecologists)  - Psychosocial support | - **Content of the oncofertility counseling** by a fertility expert (gynecologist):  - General anatomy and physiological function of the female reproductive system  - Changes in anatomy and physiological function during and after childhood cancer treatment  - Estimated personalized risk of gonadal damage  - Possible alternative family planning options including procedure, risks and benefits:   - Expectative management at this time, in the future:   - Oocyte freezing after treatment (if possible)   - Oocyte donation (from oocyte bank or family or friends)   - Intergenerational oocyte donation (oocyte cryopreservation of mothers younger than 40 years)   - Adoption - Oocyte freezing if possible - Ovarian tissue cryopreservation (OTC)   - Information on storage (including storage time)  - Explaining uncertainty on feasibility of future usage of stored tissue and success rates of achieving future pregnancies, including the current possibilities and prospects.  - Obtaining informed consent in cases that opt for ovarian tissue cryopreservation ### |
| 5. Fertility preservation (ovarian tissue cryopreservation**) | - Dedicated fertility expert (gynecologist) in close communication with pediatric oncology surgeon and involved pediatric oncologist  - Embryologist | - Choose optimal procedure for a particular patient  - Plan preservation procedure  - Timing of gonadal tissue preservation (where feasible in combination with mandatory oncological and supportive care surgery)  - Processing and freezing of ovarian tissue |

*Legend:*

*^ In our hospital this is a dedicated oncofertility nurse practitioner*

*# Communication and agreement prior to the counseling between surgeons, oncologists and gynecologists is important to ensure harmonization of thoughts on the safety, timing, feasibility and desirability of ovarian tissue cryopreservation per individual patient to avoid giving conflicting information to families.*

*## Information regarding infertility risk verbal and visual information, including flyers and information on our website.*

*### In agreement with the standard of care statement of the ASRM, 2019[1]*

** Counseling of a selected group, counseling is available for low and intermediate risk, but encouraged in high risk patients*

*** Selected subgroup of children with cancer at high risk of gonadal damage*

### **S3 Table. DCOG amended version[2] of the Edinburgh selection criteria[3] for OTC**

| **The DCOG amended Edinburgh selection criteria** |
| --- |
| A realistic chance of surviving for 5 years |
| A high risk of premature ovarian insufficiency (>50%) |
| Informed consent (from parents and, where possible, patient) |

DCOG: Dutch Childhood Oncology Group; OTC: ovarian tissue cryopreservation

<https://www.skion.nl/workspace/uploads/Consensus-fertiliteitspreservatie-mei-2016.pdf>

### **S4 Table. Characteristics of timely triaged girls organized per risk group**

|  |  | **Timely Triaged** |  |  | **Not Timely Triaged (Retrospectively)** | | |
| --- | --- | --- | --- | --- | --- | --- | --- |
|  | **Low**  **n(%)** | **Intermediate**  **n(%)** | **High**  **n(%)** | **Total**  **n(%)** | **Low**  **n(%)** | **High**  **n(%)** | **Total**  **n(%)** |
| **Diagnosis** |  |  |  |  |  |  |  |
| Hematology | **63 (41.7)** | **20 (62.5)** | **7 (15.6)** | **90 (39.5)** | **11 (39.3)** | **0** | **11 (35.5)** |
| - ALL | 31 (20.5) | 1 (3.1) | 3 (6.7) | 35 (15.4) | 8 (28.6) | - | 8 (25.8) |
| - AML | 7 (4.6) | - | 2 (4.4) | 9 (3.9) | - | - | - |
| - Other leukemia | 2 (1.3) | - | - | 2 (0.9) | 1 (3.6) | - | 1 (3.2) |
| - BMF/MDS | 2 (1.3) | - | 2 (4.4) | 4 (1.8) | - | - | - |
| - Hodgkin | 6 (4.0) | 16 (50.0) | - | 22 (9.6) | 1 (3.6) | - | 1 (3.2) |
| - NHL | 5 (3.3) | 3 (9.4) | - | 8 (3.5) | - | - | - |
| Solid tumors | **54 (35.8)** | **7 (21.9)** | **31 (68.9)** | **92 (40.4)** | **10 (35.7)** | **1 (33.3)** | **11 (35.5)** |
| - Neuroblastoma | 3 (2.0) | 2 (6.3) | 16 (35.6) | 21 (9.2) | 1 (3.6) | - | 1 (3.2) |
| - Renal tumor | 15 (9.9) | - | 1 (2.2) | 16 (7.0) | 1 (3.6) | - | 1 (3.2) |
| - Carcinoma | 2 (1.3) | - | 3 (6.7) | 5 (2.2) | 3 (10.7) | - | 3 (9.7) |
| - Osteosarcoma | 6 (4.0) | - | - | 6 (2.6) | - | - | - |
| - Ewing sarcoma | - | - | 4 (8.9) | 4 (1.8) | - | - | - |
| - Soft tissue sarcoma | 3 (2.0) | - | 5 (11.1) | 8 (3.5) | 2 (7.1) | 1 (33.3) | 3 (9.7) |
| - Germcell tumor | 11 (7.3) | 4 (12.5) | 1 (2.2) | 16 (7.0) | 3 (10.7) | - | 3 (9.7) |
| - Liver tumor | 6 (4.0) | - | 1 (2.2) | 7 (3.1) | - | - | - |
| - Skin cancer | 3 (2.0) | - | - | 3 (1.3) | - | - | - |
| - Other | 15 (9.9) | 1 (3.1) | - | 16 (7.0) | 1 (3.6) | - | 1 (3.2) |
| Neuro-oncology | **34 (22.5)** | **5 (15.6)** | **7 (15.6)** | **46 (20.2)*** | **7 (25.0)** | **2 (66.6)** | **9 (29.0)** |
| - Brain tumor | 34 (22.5) | 5 (15.6)) | 7 (15.6) | 46 (20.2) | 7 (25.0) | 2 (66.6) | 9 (29.0) |
| **Total** | **151** | **32** | **45** | **228** | **28** | **3** | **31** |

ALL: Acute Lymphoblastic Leukemia; AML: Acute Myeloid Leukemia; BMF/MDS: Bone marrow failure/ myelodysplastic syndrome; NHL: Non-Hodgkin lymphoma

*2 early deaths in neuro-oncology

**S5 Table. Gonadal damage risk stratification tool for European treatment protocols used for oncofertility care for girls in the Princess Máxima Center**

| Tumor | Protocol | Treatment arm | CED mg/m2 | Female infertility risk |
| --- | --- | --- | --- | --- |
| **Hematologic malignancies** | | | | |
| Acute Lymphoblastic Leukemia* | ALL-11 | SR, MR | 2000 | Low |
|  |  | HR 1-3 +SCT | 5600 + SCT | High |
|  |  | HR 1-6 + II | 9300 | High |
|  | Interfant06 | Germline LR/rearranged MR HR - SCT | 3000 | Low |
|  |  | Rearranged MR HR + SCT | 3000+ SCT | High |
|  | EsPhALL | Arm A | 9000 | High |
|  |  | Arm B | 3000 | Low |
|  |  | High risk arm | 3976 + SCT | High |
|  |  | High risk arm | 5976 + SCT | High |
|  | IntReALL | SR treatment arm A | 1976 | Low |
|  |  | SR treatment arm A with SCT | 1976 + SCT | High |
|  |  | SR treatment arm B | 3400 | Low |
|  |  | SR treatment arm B with SCT | 3400 + SCT | High |
|  |  | HR | 1976 + SCT | High |
|  | ALLTogether | R1 standard, experimental | 0 | Low |
|  |  | R2 standard, Exp arm A, Exp arm B | 3000 | Low |
|  |  | R3 standard, Exp InO: IR-high risk | 2000 | Low |
|  |  | ABL HR allo-SCT (≥ 1-3 NOPHO blocks) | 2000 +SCT | High |
|  |  | ABL IR-high | 2000 | Low |
|  |  | HR BCP SCT 3 blocks | 4200 +SCT | High |
|  |  | HR BCP chemo 6 blocks | 7400 | High |
|  |  | HR T-cell without Nelarabine + HR blocks | 4200 | Intermediate |
|  |  | HR T-cell with Nelarabine single | 1000 | Low |
|  |  | HR T-cell with Nelarabine single + HR blocks | 3200 | Low |
|  |  | HR T-cell with Nelarabine addition | 2000 | Low |
|  |  | HR T-cell with Nelarabine addition + HR blocks | 4200 | Intermediate |
|  |  | DS-SR | 1000 | Low |
|  |  | DS-IR, DS-HR | 3000 | Low |
| LCH | LCH IV | Stratum 1 group 1 (MS-LCH) arm A / B / C / D | 0 | Low |
| (Langerhans Cell Histiocytosis) |  | Stratum 1 group 2 (SS-LCH) | 0 | Low |
|  |  | Stratum 2 | 0 | Low |
|  |  | Stratum V without clinical neurodegeneration | 0 | Low |
|  |  | Stratum V with clinical neurodegeneration | 0 | Low |
| Hodgkin lymphoma * | EuroNet-PHL-C2 | TL1 | 1000 | Low |
|  |  | TL2 | 2000 | Low |
|  |  | TL2 intensified | 2500 | Low |
|  |  | TL3 | 4000 | Intermediate |
|  |  | TL3 intensified | 5000 | Intermediate |
| Non-B NHL (Non-Hodgkin | Euro LB-02 | T-Cell LL stage I-II | 2000 | Low |
| Lymphoma) |  | T-Cell LL stage III-IV | 3000 | Low |
|  |  | Non-T-Cell LL stage I-II | 2000 | Low |
|  |  | Non-T-Cell LL stage III-IV | 3000 | Low |
| B-NHL/B-ALL * | SKION B-NHL/B-ALL | Group A | 3000 | Low |
| (B-cell Non-Hodgkin Lymphoma/ | 2008 | Group B | 3300 | Low |
| acute lymphoblastic leukemia) |  | Group C1 | 6800 | High |
|  |  | Group C2 | 6800 | High |
|  | Inter-B-NHL ritux | Group B HR | 3300 | Low |
|  |  | Group C1 | 5800 | Intermediate |
|  |  | Group C3 | 5800 | Intermediate |
|  |  | PMLBL | 4500 | Intermediate |
| Anaplastic Large Cell Lymphoma | ALCL | LR | 3352 | Low |
|  |  | SR arm 1 SR arm 3, HR arm 1, HR arm 2, HR arm 3, HR arm 4 | 6328 | High |
| Acute Myeloid Leukemia * | Nopho DBH AML |  | without SCT | Low |
|  | 2012 |  | with SCT | High |
| Acute Promyelocytic | ICC APL 01 | SR MRD- / SR MRD+ / HR | 0 | Low |
| Leukemia | ICC APL 02 | SR, HR | 0 | Low |
| **Solid tumors** | | |  |  |
| Neuroblastoma * | DCOG NBL 2009 | OG without N4 | 0 | Low |
|  |  | OG with 1x N4 | 2100 | Low |
|  |  | OG with 2x N4 | 4200 | Intermediate |
|  |  | OG with 3x N4 | 6300 | High |
|  |  | OG with 4x N4 | 8400 | High |
|  |  | MR without N4 | 10290 | High |
|  |  | MR with N4 | 18690 | High |
|  |  | HR without N4 | 12690 | High |
|  |  | HR with N4 | 21090 | High |
|  | DCOG NBL 2009 <1yr | OG with 1x N4 <1yr | /kg | Low |
|  |  | OG with 2x N4 <1yr, 3x N4 <1yr, 4x N4 <1yr | /kg | Intermediate |
|  |  | MR without N4 <1yr, with N4 <1yr | /kg | High |
|  |  | HR without N4 <1yr, with N4 <1yr | /kg | High |
| Ewing* | Ewing 2008 | R1 female | 25176 | High |
|  |  | R3 | 25176 | High |
|  |  | R3 + TreoMel | 30776 | High |
| Osteosarcoma* | EURAMOS 1 | MAP | 0 | Low |
|  |  | MAPIE | 14640 | High |
| Renal tumors* | UMBRELLA 2016/SIOP | AV + AVD, AV + AV1, AV + AV2 | 0 | Low |
|  | 2001** | AV + HR | 8100 | High |
| Rhabdoid tumors * | EpSSG NRSTS 2005 | Cyclophosphamide | 17000 | High |
| of the kidney | EURHAB <18 mo | 3x DOX, 3x ICE, 3xVCA | 8892 | High |
| (RTK) or of soft | EURHAB <18 mo HD | 2x DOX, 2x ICE, 2x VCA + CARBO Thiotepa | 50928 | High |
| tissue (MRT) | EURHAB >18 mo | 3x DOX, 3x ICE, 3xVCA + RT | 8892 | High |
|  | EURHAB >18 mo HD | 2x DOX, 2x ICE, 2x VCA + CARBO Thiotepa + RT | 50928 | High |
| NRSTS* | EpSSG NRSTS 2005 | 3x ifosfamide | 6588 | High |
| (Non-Rhabdomyosarcoma Soft |  | 4x ifosfamide | 8784 | High |
| Tissue Sarcoma) |  | 5x ifosfamide | 10980 | High |
|  |  | 6x ifosfamide | 13176 | High |
| Soft tissue sarcomas* | EpSSG | LR subgroup A | 0 | Low |
|  | RMS2005 | SR subgroup B | 5800 | Intermediate |
|  |  | SR subgroup C (9x Ifosfamide) | 13176 | High |
|  |  | SR subgroup C (5xIfosfamide) | 7320 | High |
|  |  | SR subgroup D (9x Ifosfamide) | 13176 | high |
|  |  | HR and group A + group C | 13176 | high |
|  |  | HR and group A + group D | 17376 | High |
|  |  | HR and group B + group C | 13176 | High |
|  |  | HR and group B + group D, VHR | 17376 | High |
| Germ cell tumor | SIOP CNS GCT II | NGGCT | 7320 | High |
| Liver tumors | PHITT | Group A1 very low risk HB | 0 | Low |
| Hepatocellular |  | Group A2 very low risk HB | 0 (cisplatin) | Low |
| carcinoma |  | Group B1 Low risk HB / B2 | 0 (cisplatin) | Low |
|  |  | Group C intermediate risk SIOPEL3HR / C5VD/ CDDP-M | 0 (cisplatin) | Low |
|  |  | Group D1 high risk HB SIOPEL4, D2 high risk HB CDCE, CDVI | 0 (cis/carboplatin) | Low |
|  |  | Group E1 resected HCC | 0 | Low |
|  |  | Group E2 resected HCC PLADO | 0 (cisplatin) | Low |
|  |  | Group F unresected/metastatic PLADO sorafenib, GEMOX | 0 (cisplatin) | Low |
| **Brain tumors** |  |  |  |  |
| Opticus glioma | SIOP LGG 2004 | Vincristine, carboplatin, etoposide. (In case of allergy: cyclo) | 0 | Low |
| Intradural-extramedulary tumor | HIT-MED + SCT |  | 49500 | High |
| Medulla blastoma | SR ACNS0331 | Cyclophosphamide, lomustine | 13200 | High |
|  | HR ACNS0332 | cyclophosphamide | 12000 | High |
| AT/RT (Atypical | EURHAB <18 mo | 3x DOX, 3x ICE, 3xVCA | 8892 | High |
| teratoid/rhabdoid | EURHAB <18 mo HD | 2x DOX, 2x ICE, 2x VCA + CARBO Thiotepa | 50928 | High |
| tumors) | EURHAB >18 mo | 3x DOX, 3x ICE, 3xVCA + RT | 8892 | High |
|  | EURHAB >18 mo HD | 2x DOX, 2x ICE, 2x VCA + CARBO Thiotepa + RT | 50928 | High |
| Dysgerminoma | WHO IV SIOP CNS GCT II HR-non-germinoma | PEI | 8540 | High |
| High grade glioma, Pons glioma | ACNS0126 | Temozolamide | 0 | Unknown |
| Medulloblastoma | PNET 5 | MB-SR / MB-WNT-HR(>16years) | 17600 | High |
|  |  | MB-WNT-HR (<16years) | 13200 | High |
|  |  | MB-SHH-TP53: No alkylating agents | 0 | Low |
| HGG | Infant HGG 2013/HIT SKK | Elements IIs IIIs/1 IIIs/2 IVs | 7200 | High |

The allocated risk is based on the recently published IGHG guideline, which classifies a CED score of 6000mg/m2 as high risk. The risk allocation of patients with a renal tumor is postponed until after surgery, when the definitive treatment including radiotherapy dose is known. For patients with ALL and NHL risk allocation postponed to the moment of reaching complete remission (CR) or treatment arm allocation. Some patients with large abdominal tumors at high risk for infertility will be counseled at a later moment due to the desirability of abdominal surgery with a large abdominal tumor in situ.

*Total Body irradiation, full abdominal/pelvic radiation upgrades towards high risk. Expected unilateral removal of an ovary as part of the oncologic treatment in combination with gonadotoxic chemotherapy is also classified as high risk and OTC must be discussed.

**The UMBRELLA protocol was initiated in 2019 an prior to that the SIOP-2001 protocol was used, so both protocols were included.

### **S1 Figure. Days between start of chemotherapy and fertility preservation in patients who underwent fertility preservation**


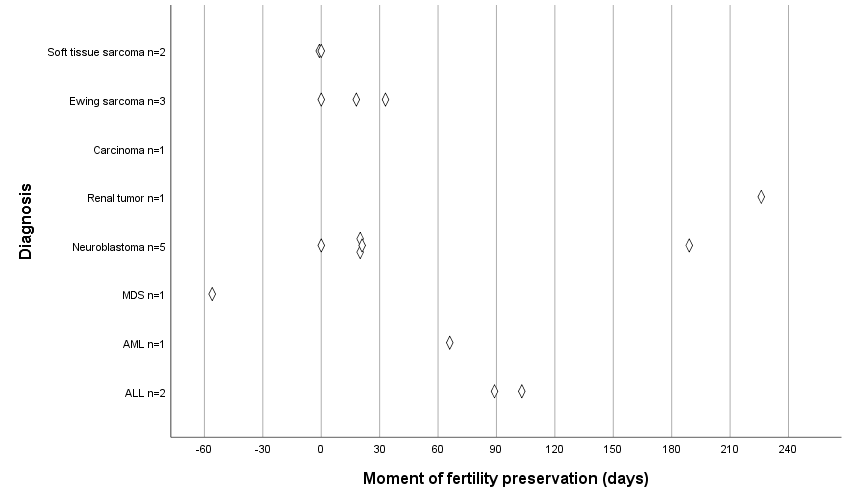


Day 0 is the start of cytotoxic therapy. MDS: myelodysplastic syndrome; AML: Acute myeloid leukemia; ALL: Acute lymphoblastic leukemia.

The patient with a carcinoma in both ovaries did not require chemotherapy and was treated only surgically.

**References:**

1. Practice Committee of the American Society for Reproductive Medicine. Electronic address aao. Fertility preservation in patients undergoing gonadotoxic therapy or gonadectomy: a committee opinion. Fertil Steril. 2019;112(6):1022-33.

2. Veening MA, Bos AME, Versluys AB, van Santen HM, van de Wetering MD, van den Heuvel-Eibrink MM, et al. SKION consensus fertiliteitspreservatie voor meisjes met kanker, van 0-18 jaar. SKION [Internet]. 2016 [cited 2020 Sept 18]. Available from: <https://www.skion.nl/workspace/uploads/Consensus-fertiliteitspreservatie-mei-2016.pdf>. Dutch.

3. Wallace WH, Smith AG, Kelsey TW, Edgar AE, Anderson RA. Fertility preservation for girls and young women with cancer: population-based validation of criteria for ovarian tissue cryopreservation. Lancet Oncol. 2014;15(10):1129-36.
